# Synergistic Charge Redistribution and Structural Evolution in Three-Dimensionally Printed Mn-Prussian Blue Analogue-Based Electrodes for Energy Storage

**DOI:** 10.1021/acsnanoscienceau.5c00167

**Published:** 2026-03-30

**Authors:** Pedro H. S. Borges, Michele V. C. O. da Silva, Jéssica S. Stefano, Rafael R. Barreto, Abner de Siervo, Eduardo M. Richter, Rodrigo A. A. Muñoz, Juliano A. Bonacin, Edson Nossol

**Affiliations:** † Institute of Chemistry, Federal University of Uberlândia, Uberlândia 38408-902, MG, Brazil; ‡ Institute of Chemistry, University of Campinas, Campinas 13083-970, SP, Brazil; § Chemistry Technology Department, Federal University of Maranhão, São Luís 65080-805, MA, Brazil; ∥ Gleb Wataghin Institute of Physics, University of Campinas,Campinas 13083-970, SP, Brazil

**Keywords:** 3D printing, fused deposition modeling, energy storage, Prussian blue analogue, electrochemical activation

## Abstract

Three-dimensional (3D) printing offers a versatile route to fabricate multifunctional electrodes by integrating key components within a single structure. Herein, we developed polylactic acid/carbon black/reduced graphene oxide/manganese hexacyanoferrate (PLA/CB/rGO/MnHCF) electrodes and evaluated their structural and electrochemical behavior to understand the cooperative dynamics governing their performance. Scanning electron microscopy and energy-dispersive X-ray spectroscopy analyses confirmed the homogeneous dispersion of MnHCF microcubes and carbonaceous additives within the PLA matrix. PLA/CB/rGO electrodes exhibited predominantly capacitive behavior, whereas MnHCF incorporation introduced distinct redox features associated with Fe^2+/3+^ and Mn^2+/3+^ transitions. Galvanostatic cycling revealed enhanced capacitance and high reversibility, with long-term testing over 3,500 cycles showing a remarkable increase in capacitance. Postcycling characterization indicated expansion of the electroactive surface area, activation of previously inaccessible MnHCF sites, and reduction of rGO/CB functional groups. Raman and FTIR analyses confirmed persistence of Mn^3+^ and Fe^3+^ centers after cycling. These findings support a cooperative mechanism in which the insulating PLA matrix imposes localized electron bottlenecks, redirecting the charge toward rGO/CB sites for reduction while sustaining MnHCF oxidation. This synergistic redistribution progressively enhances redox activity and conductivity, turning the PLA host′s insulating nature into a driver of long-term activation. Overall, the 3D-printed PLA/CB/rGO/MnHCF electrodes demonstrate stability, progressive activation, and promise for scalable and tunable energy storage applications.

## Graphical abstract

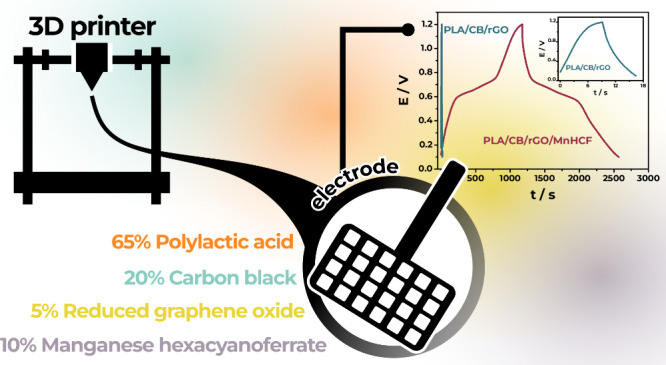

## Introduction

The growing demand for clean and efficient energy storage technologies is closely linked to the decline in fossil fuel use and the rapid expansion of electricity-based systems. To meet the requirements of a sustainable era, storage devices must combine high efficiency, long-term stability, affordability, and minimal environmental impact.^1–3^ Currently, lithium-ion batteries (LIBs) dominate the market due to their high energy density and reliable performance.^4^ However, their widespread application is limited by high costs, safety concerns, and the scarcity and toxicity of some of their components.^2,5^

Aqueous sodium-ion-based supercapacitor devices have emerged as a promising alternative to LIBs, mainly because they can deliver a high specific power density while maintaining a long cycle life. The use of an aqueous electrolyte enhances the safety and reduces the toxicity. In addition, sodium-ion systems are more cost-effective, since sodium is highly abundant in the Earth’s crust.^6,7^ Considering that the performance of energy storage devices is strongly dependent on the properties of their active materials, developing new materials with unique electrochemical characteristics remains a critical challenge.

Prussian blue (PB) has attracted considerable attention in this context.^8,9^ PB is a multifunctional compound with a zeolitic-like 3D framework composed of alternating Fe^2+^ and Fe^3+^ centers bridged by cyanide ligands.^10^ This structure enables reversible redox transitions and facilitates alkali-ion intercalation, making PB suitable for ion-intercalation-based energy storage devices.^2,11^ Moreover, replacing Fe with other transition metals yields Prussian blue analogues (PBAs) with tunable properties. Among them, manganese hexacyanoferrate (MnHCF or Mn-PBA) stands out due to its excellent performance in energy storage devices, combined with the abundance and low cost of manganese, making it a viable and sustainable option for aqueous electrolyte-based systems.^12^

Additive manufacturing has recently emerged as a promising alternative to conventional electrode fabrication, offering a low-cost and versatile route to produce complex designs.^13^ In particular, fused deposition modeling (FDM) enables the incorporation of conductive materials into printable filaments. Carbon black (CB) and reduced graphene oxide (rGO) are among the most common additives used, thanks to their high surface area, excellent electrical conductivity, and fast electron transfer.^13,14^ These properties facilitate their integration into energy storage devices. Despite recent advances, only a few studies, most notably that of Silva et al.,^15^ have demonstrated the incorporation of PBAs into FDM-based 3D-printed electrodes. This remains a challenge as the printed structures are usually dominated by insulating polymers such as polylactic acid (PLA).

Beyond the immobilization of PBAs in carbonaceous matrices,^16^ further improvements in capacitance and cycling stability can be achieved by producing defect-free, low-water-content PBAs.^17,18^ Herein, we present for the first time the fabrication of a PLA/CB/rGO/MnHCF composite electrode via FDM. Our approach involved a simple and efficient synthesis of low-defect MnHCF followed by its coating with CB and rGO supports and subsequent incorporation into a PLA matrix. The resulting 3D-printed electrode was structurally and electrochemically characterized and exhibited notable energy storage performance without the need for additional treatment. This strategy contributes to the advancement of sustainable, low-cost, and efficient energy storage devices, thereby supporting the ongoing transition toward cleaner energy technologies.

## Experimental Section

### Reagents and Solutions

All solutions were prepared with deionized water, and reagents were used as received without further purification. Carbon black (CB, VXC72) and reduced graphene oxide (rGO) were purchased from Cabot (Brazil) and CTNano/UFMG (Brazil), respectively. Polylactic acid (PLA) pellets were acquired from 3DLab (Brazil). Manganese chloride tetrahydrate (MnCl_2_·4H_2_O, > 99%) was obtained from Merck (Germany), and potassium ferricyanide (K_3_[Fe­(CN)_6_], > 99%) was from Êxodo Científica (Brazil). Sodium citrate hydrate (Na_3_C_6_H_5_O_7_·2H_2_O, > 99%) and sodium perchlorate hydrate (NaClO_4_·H_2_O, > 85%) were obtained from Panreac (Spain) and Dinâmica (Brazil), respectively.

### Instrumentation

Material morphologies were examined by scanning electron microscopy (SEM) using a Vega3 microscope (Tescan, Czech Republic) operated at 20 kV. Elemental composition was analyzed by energy-dispersive X-ray spectroscopy (EDS) with an X-Act X-ray detector (Oxford Instruments, UK) integrated into the SEM equipment. To prevent damage to the polymer components, a 10 nm gold coating was applied to the samples prior to SEM/EDS analyses. Moreover, FEG-SEM images of the electrodes before and after a long-term cycling test were acquired using a FEG-250 microscope (Quanta, USA) operated at 2.0 kV. Fourier-transform infrared spectroscopy (FTIR) was carried out on a Frontier MIR/FIR spectrometer (PerkinElmer, USA) with an attenuated total reflectance accessory (Pike Technologies, USA). Raman spectra were recorded using a LabRAM HR Evolution microscope (Horiba, Japan) with a 532 nm Ar-ion laser operated at 5% power. X-ray diffraction (XRD) patterns were obtained using a 1.54184 Å Cu X-ray source in a D8-Advance diffractometer (Bruker, USA) operated under 40 kV and 40 mA. Thermogravimetric analysis (TGA) was performed using a TGA55 Discovery (TA Instruments, USA) thermal analyzer under an air atmosphere with a temperature increase rate of 5 °C min^–1^. XPS measurements were performed in an ultrahigh-vacuum chamber (base pressure in the low 10^–9^ mbar range) using a nonmonochromated Al Kα X-ray source (*h*ν = 1486.6 eV) and a hemispherical electron energy analyzer (SPECS PHOIBOS 150). Spectra were acquired at normal emission (θ = 0°). Core-level spectra were collected using pass energies of 25 eV. The binding-energy scale was referenced by setting the C–C/C–H contribution of the adventitious C 1s signal to 284.8 eV and applying the same charge correction procedure to all samples. Core-level spectra were background-subtracted (Shirley) and fitted with a void line shape. MnHCF powder and electrode samples were mounted on conductive carbon tape in the sample holder.

### Electrochemical Measurements

Electrochemical tests were performed with a Squidstat Solo potentiostat/galvanostat (Admiral Instruments, USA). PLA/CB/rGO/MnHCF composite, Ag_(s)_/AgCl_(s)_/Cl^–^_(sat.)_, and a platinum wire served as the working, reference, and auxiliary electrodes, respectively. Measurements were conducted in a 10.0 mol L^–1^ NaClO_4_ solution under ambient air at room temperature. Prior to testing, the working electrode was conditioned by cyclic voltammetry (CV) for 15 cycles between 0 and 1.2 V vs Ag_(s)_/AgCl_(s)_/Cl^–^_(sat.)_ at 10 mV s^–1^. Electrochemical impedance spectroscopy (EIS) was performed under the OCP conditions in a 10.0 mol L^–1^ NaClO_4_ solution using a PGSTAT204 potentiostat/galvanostat (Metrohm, Switzerland). The plots were acquired between 1 kHz and 10 mHz using a 10 mV potential amplitude. For the electrochemical tests, the electrode was dipped in solution until the very beginning of its stem. More details of the electrode design are presented in the following topics.

### Mn-PBA Preparation

Two aqueous solutions (1.5 L each) were freshly prepared: (A) 10.0 mmol L^–1^ of K_3_[Fe­(CN)_6_] and (B) 10.0 mmol L^–1^ of MnCl_2_·4H_2_O with 15.0 mmol L^–1^ of sodium citrate. Solution B was stirred vigorously, while solution A was added dropwise at 25 mL min^–1^. During addition, the Mn^2+^ solution gradually changed from light pink to orange and finally to a turbid brown suspension, indicating nucleation and growth of MnHCF particles. After the addition, the suspension was kept in a hot-water bath at 80 °C for 12 h. The solid gradually turned to an off-white color, consistent with the filling of ferricyanide vacancies in the MnHCF framework.^17^

### Filament Preparation

A conductive filament was prepared with a PLA:CB:rGO:MnHCF ratio of 65:20:5:10 (w/w) using 13.0 g of PLA, 4.0 g of CB, 1.0 g of rGO, and 2.0 g of MnHCF, resulting in a final weight of 20.0 g. First, CB, rGO, and MnHCF were mixed in 120 mL of a 1:1 water-acetone solution and stirred vigorously for 24 h to promote the coating of MnHCF with carbonaceous materials. The dispersion changed from heterogeneous black and white to a uniform dark gray. The mixture was dried at 80 °C for 48 h, and the resulting powder was blended with PLA pellets by manual mixing at 200 °C for 1 h, producing solid agglomerates. These were manually ground and extruded in a Filmaq3D extruder (Brazil) at 210 °C to obtain PLA/CB/rGO/MnHCF filaments with diameters of 1.75 mm.

### Electrode 3D Printing

Electrodes were designed in SketchUp Pro 2021 (USA), sliced using FlashPrint 5 (Brazil), and printed on a Creator Pro 2 3D printer (Brazil). The design dimensions are shown in Figure 1a and the sliced model is shown in Figure 1b. Printing conditions were a layer height of 0.2 mm, one shell, grid infill with 86% density, printing speed of 10 mm s^–1^, extruder temperature of 230 °C, and bed temperature of 70 °C. Five printed electrodes had an average mass of 59.5 ± 6.1 mg, with representative examples presented in Figure 1c.

**Figure 1:**
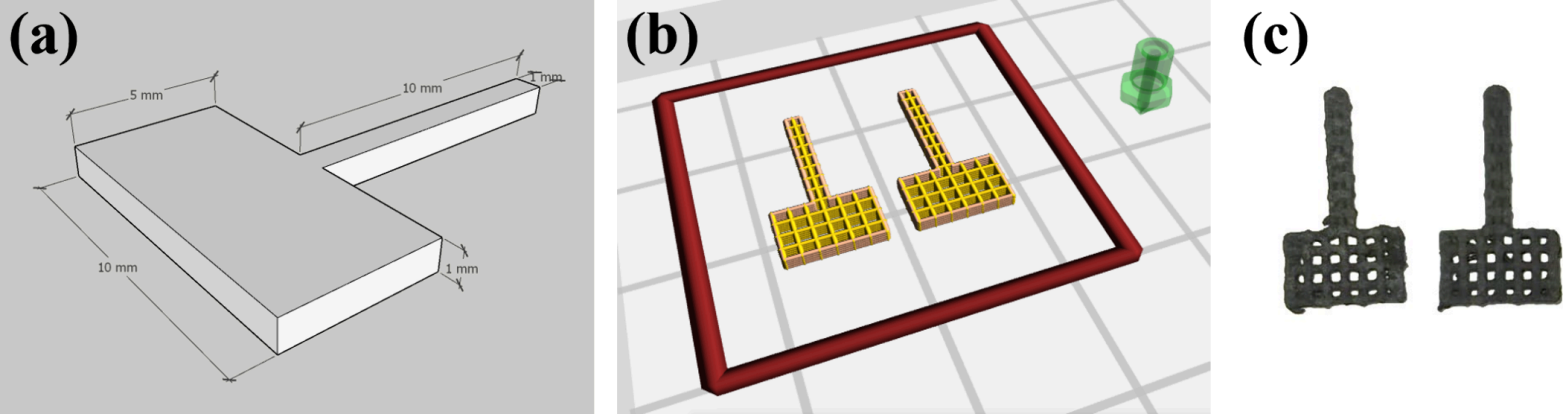
Design dimensions (a), slicing model (b), and representative 3D-printed PLA/CB/rGO/MnHCF electrodes (c).

## Results and Discussion

### Characterization of the Components

The components of the 3D-printed electrode were analyzed individually by SEM. PLA (Figure S1a) showed a smooth polymer surface with irregular microfragments. CB (Figure S1b) exhibited a highly porous texture formed by carbon nanoparticles, indicative of a large surface area. rGO exhibited crumpled, sheet-like structures with wrinkles typical of this bidimensional material (Figure S1c). Mn-PBA microcubes (Figure S 1d) were formed from smaller cubic particles, consistent with defect repair during thermal aging. Collections of well-shaped cubic particles are shown in Figure S1e. Particle size analysis of 400 microcubes (Figure S1f) yielded an average dimension of 1.98 ± 0.68 μm. EDS spectrum (Figure S2) confirmed the expected elements of MnHCF (C, N, K, Mn, and Fe), along with Si and O from the substrate. The elemental ratio K:Mn:Fe was 1.36:1.0:0.93, consistent with a low-vacancy Mn-PBA, in agreement with literature reports.^19,20^

FTIR spectra of the electrode components are shown in Figure S3a. PLA exhibited characteristic polymer vibrations (Table S1) along with additional bands at 1559, 1636, and 2849 cm^–1^ attributed to C=CH_2_ and C=C stretching, which are indicative of chain degradation by thermal, hydrolytic, or photochemical processes.^21–23^ The damaged surface observed by SEM may correlate with these degradation products. CB showed three main bands at 1068, 1537, and 1704 cm^–1^, corresponding to C–O–C, C=C, and C=O stretching, respectively, features characteristic of oxidized amorphous carbon.^24,25^ rGO displayed bands at 1042 and 1173 cm^–1^ (epoxy groups), 1550 cm^–1^ (graphitic sp^2^ carbon), and 1739 cm^–1^ (carbonyl/carboxyl groups). The prominent sp^2^-carbon band reflects partial restoration of conductivity, while residual oxygen groups indicate incomplete reduction.^26,27^ MnHCF exhibited bands at 449 and 590 cm^–1^ (Fe–CN bending and Fe–C stretching, respectively) and strong band at 2056 cm^–1^, assigned to C≡N stretching vibrations bridging the metal centers.^20,28^

Raman spectra collected with a 532 nm laser are presented in Figure S3b. PLA showed its characteristic vibrational modes (Table S2).^29^ CB and rGO displayed typical carbonaceous D, G, and 2D bands. The G band corresponds to in-plane vibrations of sp^2^ carbon atoms, while the D band reflects structural defects. The 2D band provides information about layer stacking: *A*_2D_/*A*_G_ ratios of 0.36 (CB) and 0.52 (rGO) indicate multilayered structures, with CB showing greater stacking. The *A*_D_/*A*_G_ ratio revealed significant defect densities: 4.05 for CB and 3.36 for rGO. The higher value for CB reflects amorphous disorder, whereas rGO defects are mainly attributed to residual oxygen groups and defective carbon rings.^26,27^ MnHCF displayed two cyano-bridge vibrational bands at 2085 and 2124 cm^–1^, corresponding to Fe^2+^–C≡N–Mn^2+^ linkages and assigned to E_g_ and A_1g_ modes.^30,31^ These features confirm that MnHCF is predominantly composed of reduced Fe^2+^ and Mn^2+^ centers.

### Electrode Characterization

SEM images of electrodes produced from PLA/CB/rGO/MnHCF (65:20:5:10) and PLA/CB/rGO (75:20:5) filaments are shown in Figure 2 and Figure S4, respectively. The PLA/CB/rGO electrode (Figure S4a,b) exhibited a smooth yet rugose surface, suggesting good homogeneity between the carbonaceous fillers and the polymer matrix. SEM images of the PLA/CB/rGO/MnHCF electrode are shown in Figure 2a,b. The individual components are indicated by arrows in Figure 2a, demonstrating their good dispersibility along the material’s matrix. In comparison to Figure S1d, the components did not show any morphological alterations passing through high-temperature mixing, extrusion, and printing processes. The well-mixed CB nanoparticle-incorporated PLA matrix is clearly decorated with dispersed two-dimensional rGO sheets, as well as the repaired MnHCF cubic particles, suggesting that the morphological features of the electrode’s constituents are conserved.

**Figure 2:**
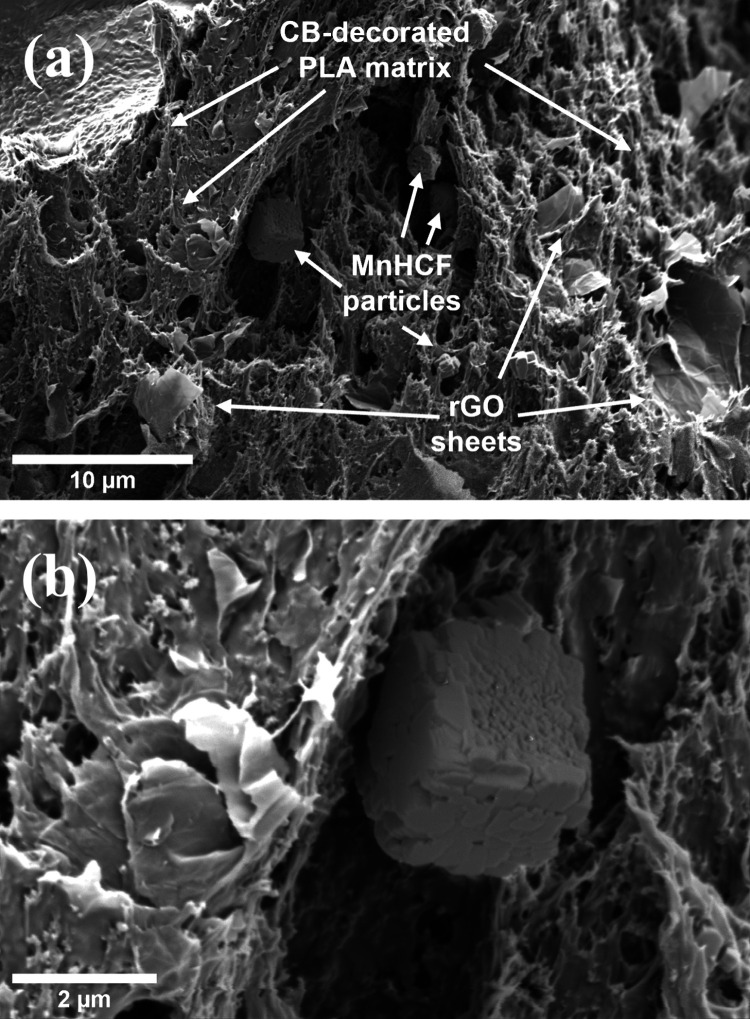
SEM images of the PLA/CB/rGO/MnHCF 3D-printed electrode.

EDS mapping (Figure S5) showed the elemental distribution along the PLA/CB/rGO/MnHCF electrode. As observed, carbon, which is the major element of PLA, CB, and rGO components, is presented as finely dispersed along the measured area, representing the polymer and carbonaceous materials matrices. Differently, potassium, iron, and manganese are highlighted in brighter spots, randomly dispersed, and in the form of aggregates. This suggests that the manual mixing is able to homogeneously distribute the components, guaranteeing good electrode manufacture reproducibility.

TGA was performed in order to evaluate potential decomposition of the electrode’s components under the high temperature employed in their preparation process. Figure S6a shows the thermal behavior of MnHCF and PLA/CB/rGO/MnHCF until 260 °C. The decreasing curve from 20 to 120 °C indicates the removal of adsorbed hydration water molecules for both materials. Typically, PBAs possess strongly coordinated water molecules as part of their structure. The repairing process employed in the MnHCF synthesis promote the reconstruction of defective sites occupied by bound water species, resulting a very low coordinated water molecules content (<1% in weight), preserving the structural arrangement until 240 °C. This suggests MnHCF structure is not affected by the high-temperature procedures. At that temperature range, PLA/CB/rGO/MnHCF electrode composition was barely influenced, resulting only in dehydration events, demonstrating good thermal stability at the high temperature electrode preparation conditions.

XRD of the MnHCF and PLA/CB/rGO/MnHCF materials is presented in Figure S6b. The MnHCF diffractogram revealed a crystalline monoclinic phase with symmetry categorized in the *P21/n* space group. No phase changes, lattice expansion/compression, or loss of crystallinity was observed when the PBA was incorporated into the polymer/carbon matrices. In addition, the presence of a broad peak centered between 17.0 and 30.0° can be associated with the presence of amorphous carbon-based and PLA materials.^32–34^ This attests to no structural changes during the production of the 3D-printed electrodes after several high-temperature procedures. High-resolution XPS was performed to evaluate the oxidation states of the metal centers of the MnHCF, and the spectra are shown in Figure S6c,d. The Mn 2p_3/2_ spectrum revealed the presence of both reduced and oxidized Mn species, while the Fe 2p_3/2_ spectrum exhibited the predominance of the Fe^3+^ oxidized state. This suggests the presence of mixed valence Fe^3+^–C≡N–Mn^2+/3+^ moieties in the PBA composition.

FTIR spectra of the electrodes are shown in Figure 3a. Characteristic PLA vibrational modes (orange marks) were clearly detected, consistent with its role as the major component. The absence of band shifts in PLA-related signals indicates that the polymer functions mainly as a structural support without strong interactions with the fillers. rGO vibrational bands (yellow marks) exhibited slight shifts, particularly the carbonyl stretching mode (1742 cm^–1^ in PLA/CB/rGO, 1743 cm^–1^ in PLA/CB/rGO/MnHCF, and 1739 cm^–1^ in isolated rGO), suggesting interactions with rGO/CB functional groups. The cyano stretching band of MnHCF appeared at 2060 cm^–1^, slightly shifted from 2056 cm^–1^ in the isolated PBA, confirming the presence of the component in the electrode.^30,31^

**Figure 3:**
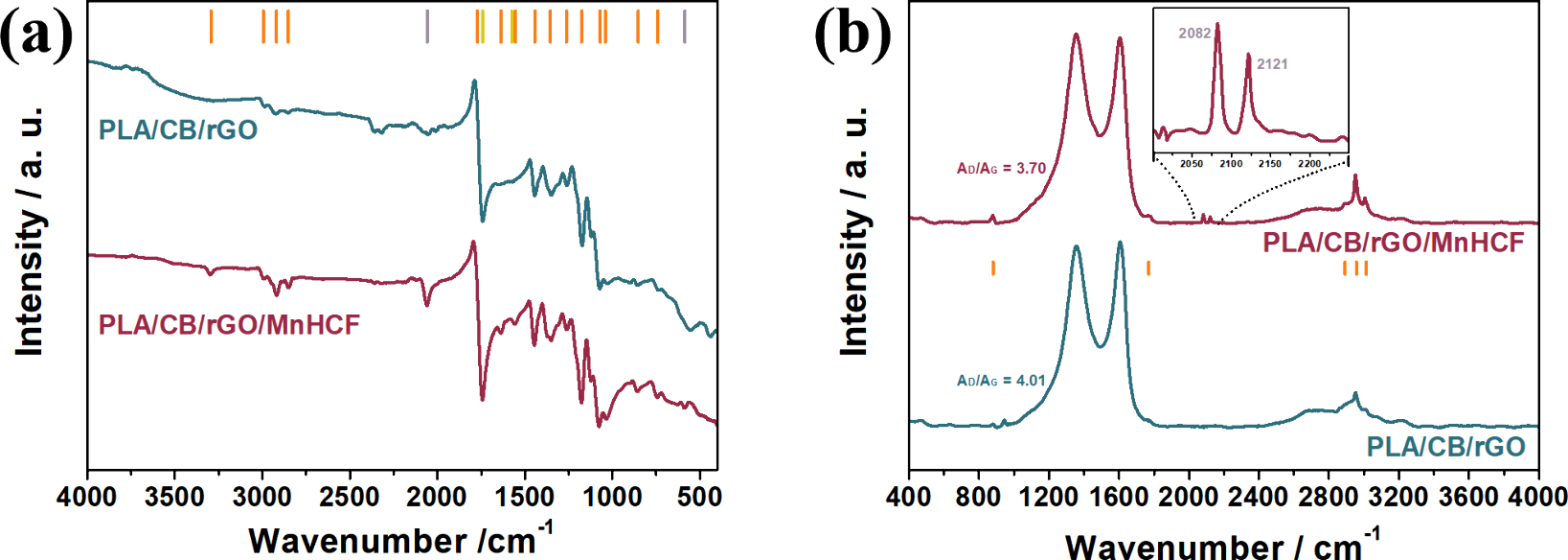
FTIR (a) and Raman (b) spectra of the PLA/CB/rGO and PLA/CB/rGO/MnHCF electrodes.

Raman spectra of both electrodes are listed in Figure 3b. PLA signals were again evident (orange marks). Carbonaceous D, G, and 2D bands showed no wavenumber shifts, indicating that MnHCF did not alter the stacking interactions within the sp^2^-carbon framework. The PLA/CB/rGO electrode displayed an *A*_D_/*A*_G_ ratio of 3.70, intermediate between the values for isolated rGO (3.36) and CB (4.05), consistent with a mixed carbonaceous matrix. Incorporation of MnHCF increased the *A*_D_/*A*_G_ ratio to 4.01, suggesting the formation of additional defects in the carbon network.^27^ MnHCF vibrational modes were detected at 2082 and 2121 cm^–1^, corresponding to the E_g_ and A_1g_ modes of the Fe^3+^–C≡N–Mn^2+^ moieties. Compared to isolated MnHCF, these bands exhibited minor shifts, reflecting interactions with the polymer-carbon matrix.^30,31^

### Electrode Performance

Before electrochemical testing, the electrodes were conditioned by cyclic voltammetry (CV) at 10 mV s^–1^ in 10 mol L^–1^ NaClO_4_ solution for 15 cycles (Figure S7). NaClO_4_ concentrated electrolyte was chosen due to the high salt solubility. Moreover, ClO_4_^–^ anions promote poor adsorption in the PBA structure, favoring their structural conservation under several electrochemical stimulations.^35^ The stability is improved by the highly concentrated NaClO_4_ electrolyte once it allows the intercalation of desolvated Na^+^ ions with a reduced hydration shell, which would compromise the MnHCF framework.^36^ The CV profiles from 0 to 1.2 V at 1 mV s^–1^ revealed distinct electrochemical behaviors. As shown in Figure 4a, the PLA/CB/rGO/MnHCF electrode displayed two redox pairs: Ia (0.693 V) and Ic (0.569 V), corresponding to Fe^2+^/Fe^3+^ transitions, and IIa (0.734 V) and IIc (0.643 V), attributed to Mn^2+^/Mn^3+^ redox activity.^37^ In contrast, the PLA/CB/rGO electrode exhibited a nearly capacitive profile with no evident faradaic contributions. Consistent with these results, galvanostatic charge–discharge (GCD) tests at 0.5 mA g^–1^ (Figure 4b) showed a purely capacitive response for PLA/CB/rGO, while PLA/CB/rGO/MnHCF exhibited charging and discharging plateaus associated with the redox processes and Na^+^ intercalation into the MnHCF framework, as demonstrated by eqs 1 and 2. This confirms the significant role of PBA incorporation in enhancing the charge storage capability.

$${\mathrm{Na}}_{2}{\mathrm{Mn}}^{\mathrm{II}}[{\mathrm{Fe}}^{\mathrm{II}}(\mathrm{CN}{)}_{6}]\cdot xH_{2}O⇌{\mathrm{NaMn}}^{\mathrm{II}}[{\mathrm{Fe}}^{\mathrm{III}}(\mathrm{CN}{)}_{6}]\cdot xH_{2}O+{\mathrm{Na}}^{+}+e^{‐}$$ 1

**Figure 4:**
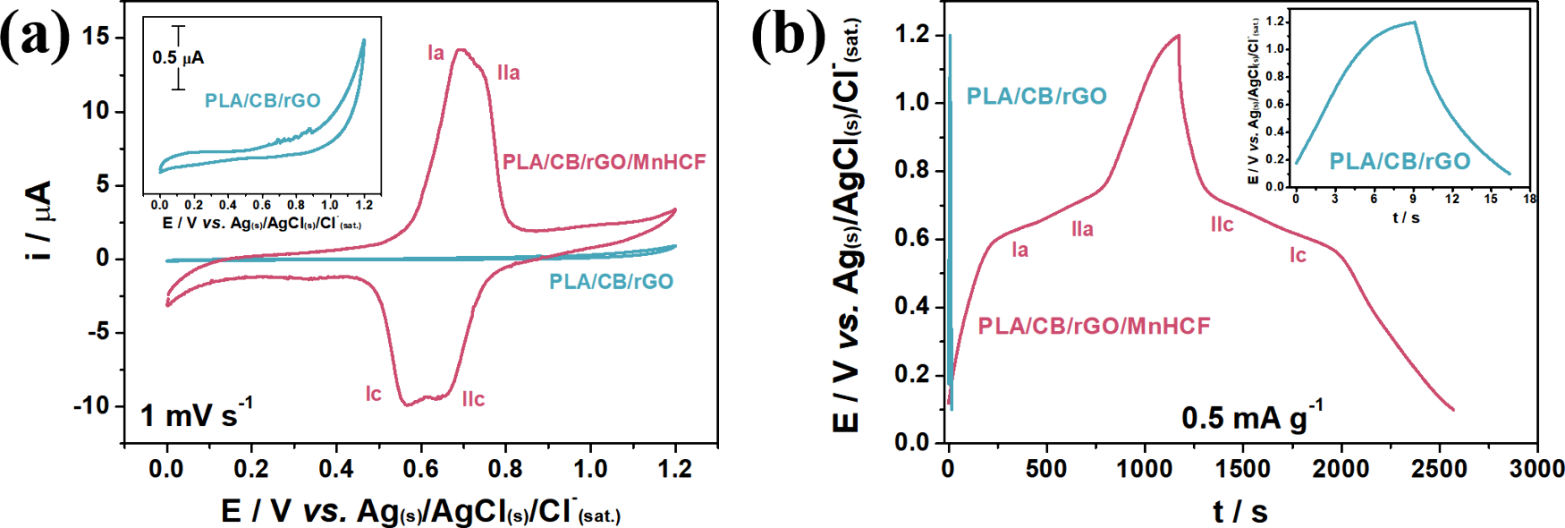
CV (a) and GCD (b) curves of PLA/CB/rGO and PLA/CB/rGO/MnHCF electrodes in a 10.0 mol L^–1^ NaClO_4_ electrolyte solution.

$${\mathrm{NaMn}}^{\mathrm{II}}[{\mathrm{Fe}}^{\mathrm{III}}(\mathrm{CN}{)}_{6}]\cdot xH_{2}O⇌{\mathrm{Mn}}^{\mathrm{III}}[{\mathrm{Fe}}^{\mathrm{III}}(\mathrm{CN}{)}_{6}]\cdot xH_{2}O+{\mathrm{Na}}^{+}+e^{‐}$$ 2

To further investigate the charge storage mechanism, CV scans were conducted at different scan rates (1–100 mV s^–1^) for PLA/CB/rGO/MnHCF (Figure 5a). Analysis using Dunn’s method revealed that a 1 mV s^–1^, diffusion-controlled process dominated (77.7%), reflecting sufficient time for semi-infinite Na^+^ diffusion into the MnHCF structure (Figure 5b). At 100 mV s^–1^, the capacitive contribution prevailed (77.3%), indicating pseudocapacitive surface-confined redox reactions (Figure 5c). The progressive increase in capacitive contribution with scan rate is summarized in Figure 5d, highlighting the transition from bulk-diffusion intercalation at slower scan rates to surface-limited kinetics at higher ones.

**Figure 5:**
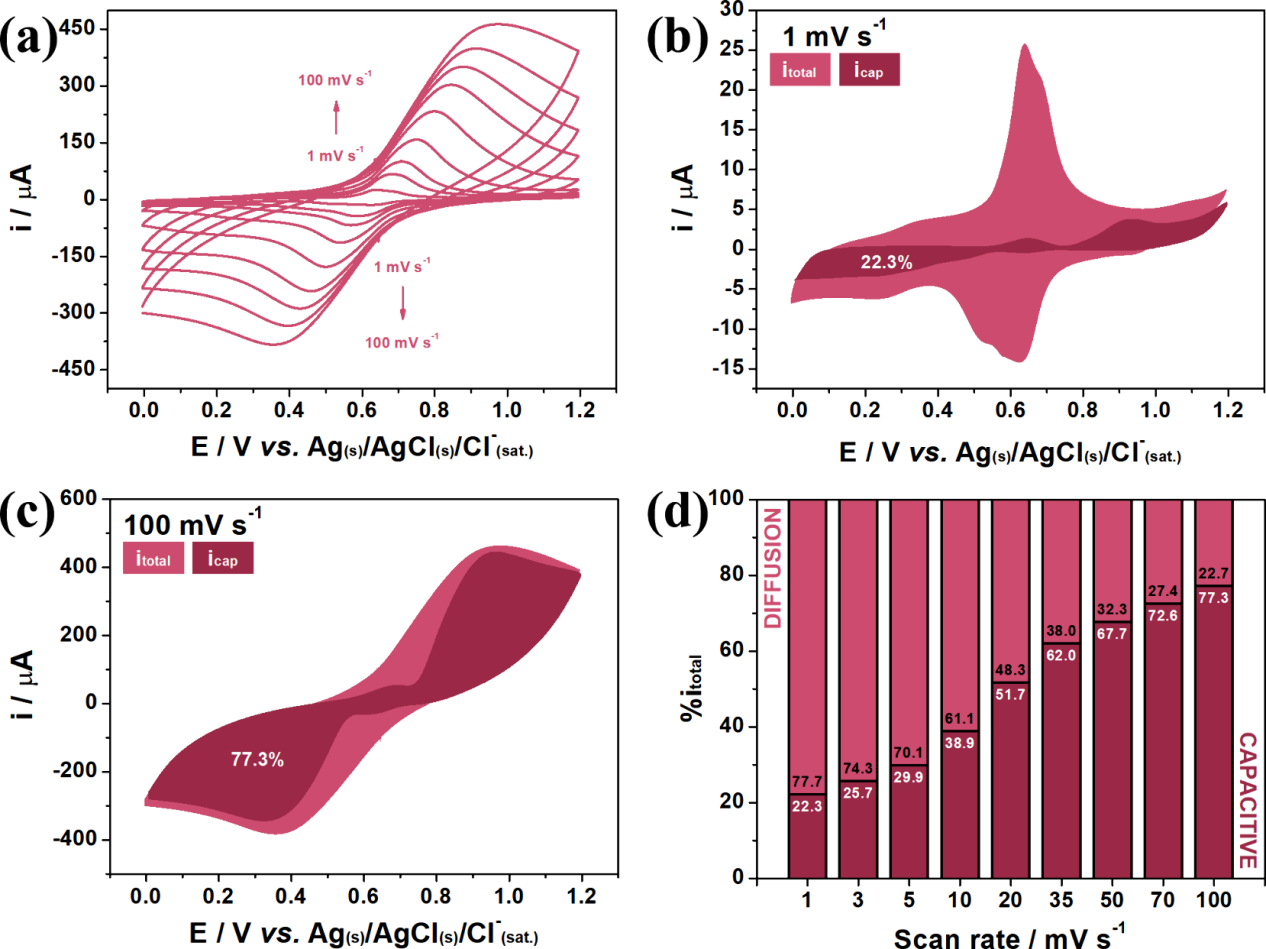
CV scan rate test of the PLA/CB/rGO/MnHCF electrode in 10.0 mol L^–1^ NaClO_4_ electrolyte solution (a). CVs with current contributions calculated by Dunn’s method of the electrode at 1 mV s^–1^ (b) and 100 mV s^–1^ (c). Summary of current contributions across all scan rates (d).

The rate capability of the PLA/CB/rGO/MnHCF electrode was evaluated by GCD under various current densities in 10.0 mol L^–1^ NaClO_4_ (Figure 6a). The electrode maintained good reversibility across all current densities, from 0.1 to 10 mA g^–1^, corresponding to discharge rates between around of approximately 2.7 and 2,500C. A maximum discharge capacitance of 132.8 mF g^–1^ was obtained at 0.1 mA g^–1^, decreasing to 15.0 mF g^–1^ at 10 mA g^–1^ (Figure 6b).

**Figure 6:**
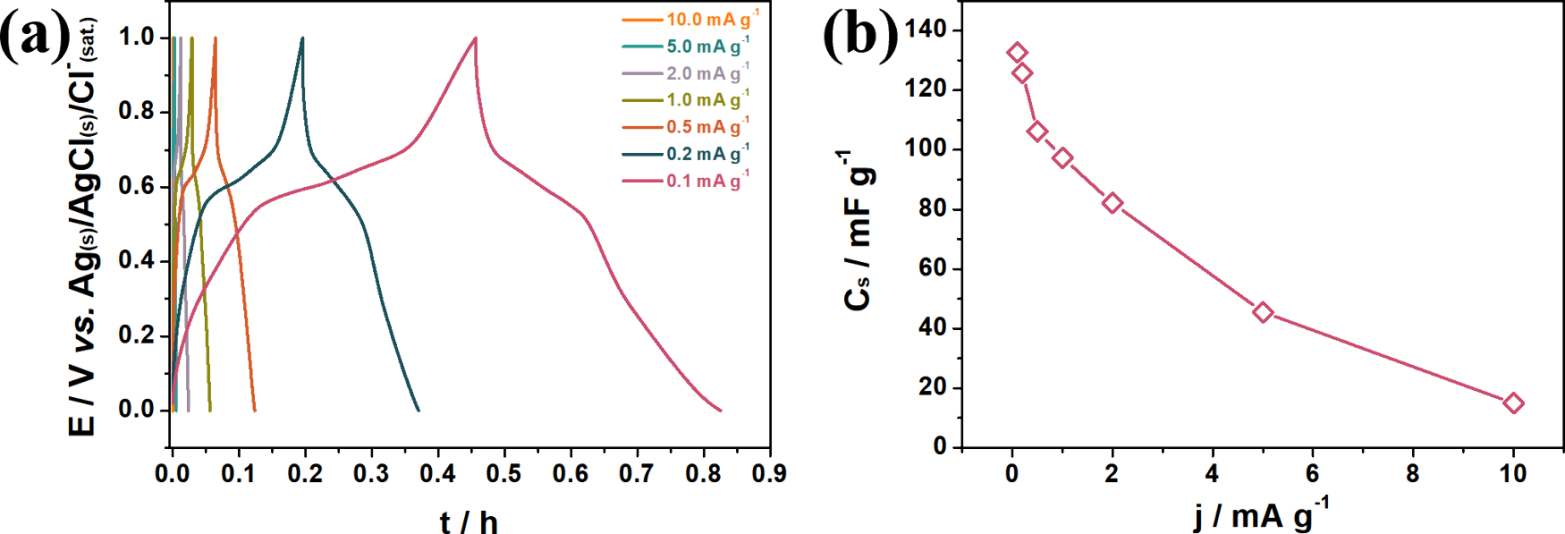
GCD profiles of the PLA/CB/rGO/MnHCF electrode in 10.0 mol L^–1^ NaClO_4_ electrolyte solution (a) and corresponding discharge capacitances at different current densities (b).

Long-term cycling stability was assessed over 3,500 GCD cycles at 0.5 mA g^–1^ (Figure 7a). The electrode exhibited an average Coulombic efficiency of 96.3% and a gradual increase in capacitance during cycling. Remarkably, the discharge capacitance rose to 247.9 mF g^–1^ after 3,500 cycles, approximately 2.5 times the initial value (Figure 7b). The pre- and postcycling specific capacitances across different current densities are compared in Figure 7c, showing a ∼250% increase (106.1 to 269.2 mF g^–1^) on average. Such activation is commonly attributed to the progressive exposure of electroactive sites in CB, rGO, and MnHCF.^38,39^

**Figure 7:**
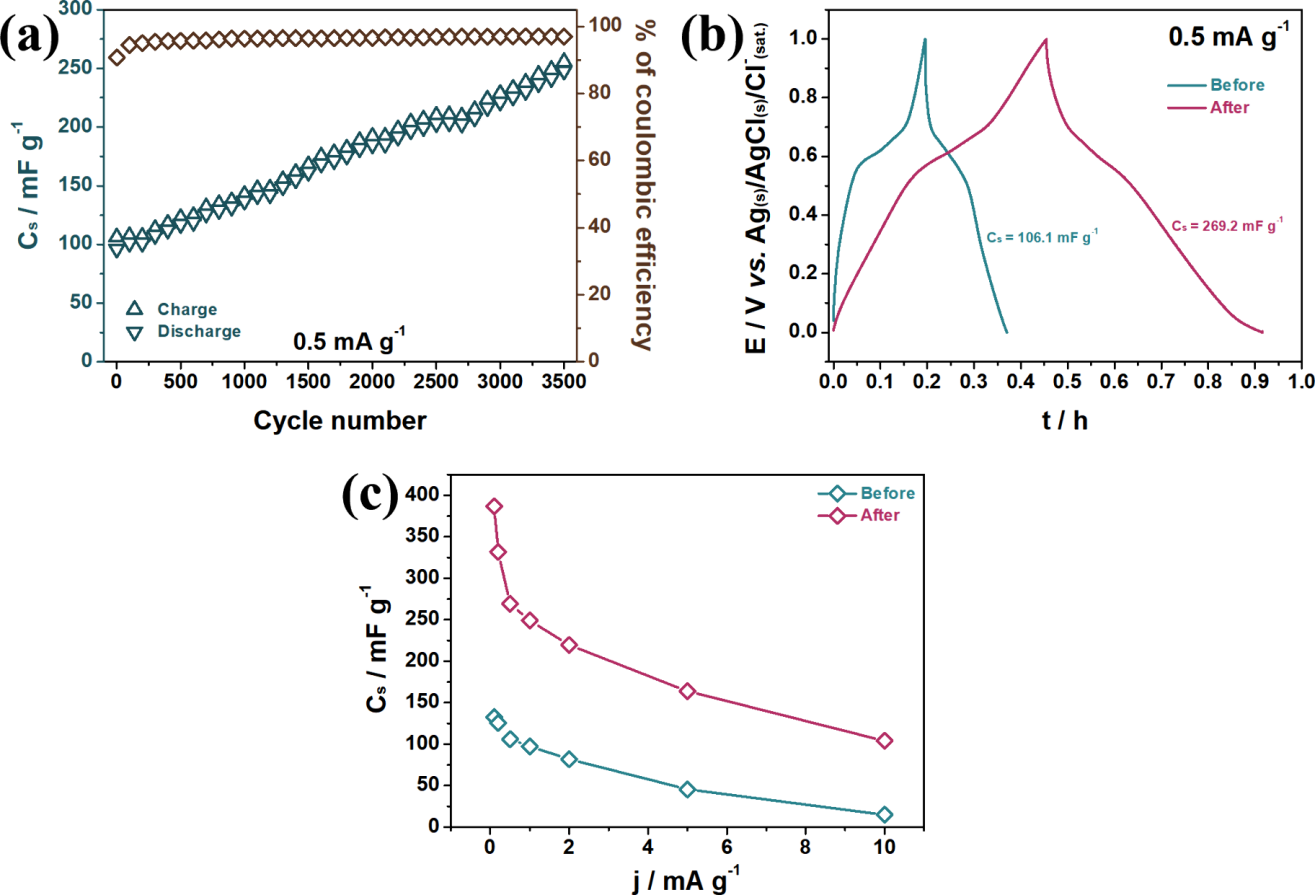
Long-term cycling performance of the PLA/CB/rGO/MnHCF electrode over 3,500 GCD cycles in 10.0 mol L^–1^ NaClO_4_ electrolyte solution (a). GCD profiles (b) and discharge capacitances at different current densities (c) before and after long-term cycling.

Postcycling analyses were carried out to elucidate these improvements. CV of the cycled electrode (Figure S8a) revealed an enlarged area, particularly in the capacitive region, suggesting the gradual activation of new electroactive surfaces. The MnHCF redox peaks also became more intense, reflecting the greater accessibility of PBA sites. Dunn’s analysis confirmed a higher diffusion-controlled current contribution compared to the fresh electrode (Figure S8b,c), consistent with extended ion diffusion pathways formed during cycling.^40,41^ SEM images of the PLA/CB/rGO/MnHCF electrode after a 3,500 cycle stability test are shown in Figure S9. It is observed that MnHCF particles did not show any loss of their original cubic morphology aspects (Figure S9a), suggesting that long-term cycling conserves particle integrity of the PBA. In addition, Figure S9b reveals larger exposition of the conductive carbon materials, as shown by the increase in rugosity promoted especially by CB nanoparticles, which contributes to the capacitance enhancement observed in the postcycling electrochemical tests.

EIS was performed before and after 3,500 GCD cycles in a 10.0 mol L^–1^ NaClO_4_ solution from 1 kHz to 10 mHz under 10 mV potential amplitude in OCP conditions. The Nyquist diagram revealed clear differences in the PLA/CB/rGO/MnHCF electrode, as shown in Figure S10a. Both diagrams were well fitted (χ^2^ ≈ 0.01) by using a [*R*(*RQ*)*Q*] equivalent circuit. First, a noticeable reduction in solution resistance (*R*_s_) from 2973.1 to 1092.6 Ω was observed, suggesting an electroactive enlargement after continuous cycling.^42^ Also, a huge drop in charge transfer resistance (*R*_ct_) from 3838.2 to 264.7 Ω revealed easier accessibility of the electrolyte, promoting more effective contact of the conductive fraction with the interface. Double layer capacitance, represented by the constant phase element 1 (CPE_1_), showed an increasing trend from 40.1 to 314 μΩ^–1^ s^N^, with the *N* factor decreasing from 0.67 to 0.44 after long-term cycling. This effect can be attributed to a higher surface area in the interface, promoted by the electrolyte percolation and contact in a more rugous carbon-based matrix.^43^ The same trend is observed in CPE_2_, which is associated with the electrolyte interface with repaired MnHCF particles. The increase from 0.63 to 3.86 mΩ^–1^ s^*N*^, where *N* is 0.7 and 0.87, respectively, indicates ionic penetrability in the low-defect MnHCF framework, creating a more homogeneous interface distribution. Those results suggest that long-term cycling gradually promotes PLA/CB/rGO/MnHCF activation through electrolyte percolation, allowing effective ionic access into the structure and optimizing the interface contact between Na^+^ ions and conductive fractions of the electrode.

Postcycling XRD of the PLA/CB/rGO/MnHCF electrode (Figure S10b) revealed the absence of additional peaks in MnHCF monoclinic pattern, attesting to no phase changing after the long-term GCD test. In addition, no considerable peak enlargement was observed after the test; thus, the crystalline nature of the MnHCF was conserved. Moreover, a slight 0.14° peak shifting to lower 2θ indicates lattice volume expansion after continuous insertion and extraction of Na^+^ ions, which was also observed in previous reports.^44,45^ The FTIR spectrum of the PLA/CB/rGO/MnHCF after 3,500 GCD cycles is shown in Figure S10c. New bands appeared and are indicated by asterisk marks. Two orange marks at 3500 and 1635 cm^–1^ are associated with OH vibrational modes resulted from the percolated electrolyte after long time immersion in highly concentrated NaClO_4_ solution.^46^ The emerging band around 2300 cm^–1^ (black asterisk) is attributed to electrode-trapped CO_2_, which can be derived from reduction products of the oxygen functional groups present in carbon materials.^47^ A rising band at 2122 cm^–1^, indicated by the lilac asterisk, can be assigned to the C≡N stretching mode bound to higher oxidation state metal centers in the MnHCF structure, i.e., Mn^3+^/Fe^3+^.^48,49^

Raman spectra of the PLA/CB/rGO/MnHCF electrodes before and after the long-term cycling test are presented in Figure S10d. Postcycling material presented the typical carbon-related D and G bands with a lower *I*_D_/*I*_G_ ratio of 2.89. This difference indicates the reduction of oxygen functional groups present in the carbonaceous components, restoring sp^2^ carbon domains, and contributing to the enhancement of conductivity of the electrode.^47^ Also, bands associated with the ClO_4_^–^ anions are highlighted, suggesting an entrapped electrolyte, which could contribute to the enrichment of the unit cell with Na^+^ and expanding MnHCF lattice. In addition, OH vibrational mode was detected, attributed to the electrolyte percolation into the electrode matrix. Furthermore, the inset shows alterations in C≡N vibrational modes of the MnHCF. Besides the shifting of the A_1g_ and E_g_ modes to higher wavenumbers, which suggests stronger interactions of the PBA with the polymer/carbon matrices, a band at 2172 cm^–1^ was raised. This CN-associated band is normally present in coordination environments with higher oxidation state Mn^3+^/Fe^3+^ centers, confirming irreversible oxidation of the metal sites, as also observed in FTIR measurements.^49^

High-resolution XPS of the PLA/CB/rGO/MnHCF electrode before and after the stability test was acquired in the C 1s and O 1s regions, as shown in Figure S11a,b, respectively. As constituted by 65% PLA, surface-sensitive XPS detected no significant alterations in the C–C, C–O, O=C–O, and C–M bonds (Figure S11a), guaranteeing no degradation of the polymeric matrix. Furthermore, differences in C–O/C=O ratios could indicate slight reduction of C–O functional groups of the carbonaceous components (Figure S11b). Although it was not possible to detect measurable Mn 2p_2/3_ and Fe 2p_2/3_ regions, previously presented characterization results sustain their predominant presence in oxidized metal states. Together, these results indicate that repetitive GCD cycling triggers MnHCF oxidation and the concomitant reduction of rGO/CB functional groups,^50^ leading to a synergistic evolution of the electrode structure. The reduction of rGO/CB is evidenced by the progressive enlargement of the CV area, consistent with restoration of the sp^2^ carbon network and improved charge transport.^51^ Simultaneously, the increasing intensity of MnHCF reduction peaks demonstrates that a greater fraction of the Mn^3+^ and Fe^3+^ centers becomes electrochemically accessible during cycling. This interpretation is further corroborated by postcycling Raman spectroscopy and FTIR analyses, confirming that the high-valence redox centers continue to actively participate in charge storage even after prolonged cycling.

The insulating nature of PLA, while limiting direct electron transport, may act as a localized barrier that hinders the transfer of MnHCF-donated electrons to the current collector. Under these conditions, nearby rGO/CB oxygenated moieties serve as alternative electron sinks, undergoing reduction and progressively restoring their conductivity. Although the 3D-printed PLA/CB/rGO/MnHCF possesses an inferior electrochemical performance when compared to other electrode preparation methods presented in the literature (Table 1), FDM techniques allows low-cost, large-scale production of functional electrode materials. Furthermore, simple raw material preparation and an ample range of electrode geometry possibilities are strong advantages, providing expansion of differently designed types of operational devices.^52–54^ This work opens new opportunities for FDM-based functional electrode manufacturing, supporting novel research regarding the production of high-performance energy storage devices.

**Table 1:** PBA-Based Energy Storage Components: Manufacture Methods and Performances^a^

material	electrode production	specific capacity/capacitance	cyclability	ref
**PLA/CB/rGO/MnHCF**	FDM	269.2 mF g^–1^ (0.5 mA g^–1^)	3,500	this work
**FNCG-PLA**	FDM	17.52 mAh g^–1^ (8 mA g^–1^)	200	^15^
**PLA/Gr/NiHCF**	FDM	37.33 mF cm^–2^ (0.1 mA cm^–2^)	2,000	^43^
**CoHCF ink**	DIW	142.4 F g^–1^ (1 A g^–1^)	15,000	^55^
**NiHCF/CNT**	drop-casting	222.95 C g^–1^ (1 A g^–1^)	5,000	^56^
**CeCoHCF**	slurry	268.8 mAh g^–1^ (1 A g^–1^)	1,000	^57^
**CuFe-PBA**	slurry	220 F g^–1^ (1 A g^–1^)	10,000	^58^
**Zn-PBA**	slurry	36 F g^–1^ (0.5 A g^–1^)	-	^59^

a **FNCG-PLA**: FeHCF-Nb_2_O_5_–CB-rGO-PLA; **PLA/Gr/NiHCF**: PLA-graphite-NiHCF; **DIW**: direct ink writing.

## Conclusions

This work demonstrates the effective fabrication of PLA/CB/rGO/MnHCF electrodes via 3D printing, enabling the seamless integration of insulating, conductive, and redox-active components within a single composite architecture. Initial tests revealed a specific capacitance of up to 132.8 mF g^–1^ at a 0.1 mA g^–1^ current density. Remarkably, despite the high PLA content, the electrodes exhibited progressive electrochemical activation over 3,500 charge–discharge cycles, leading to the formation of new ion diffusion pathways and enhanced redox performance. This behavior is attributed to a cooperative mechanism in which MnHCF oxidation is dynamically coupled with reduction of rGO/CB functional groups, likely facilitated by localized electron blockage imposed by the PLA matrix. The internal redistribution of charge carriers restores conductivity and increases redox accessibility over time, achieving a specific capacitance of 269.2 mF g^–1^ at 0.5 mA g^–1^ current density after 3,500 GCD cycles. Overall, these findings highlight the untapped potential of 3D-printed polymer-carbon-PBA composites as highly durable, tunable, and cost-effective platforms for next-generation energy storage technologies. The approach offers a promising route for designing multifunctional electrodes tailored for sustainable and scalable applications in novel electrochemical devices.

## Supplementary Material



## Notes

SEM images of PLA, CB, rGO, and MnHCF and their particles size histogram distribution. EDS of the MnHCF particles. FTIR and Raman spectra of the PLA/CB/rGO/MnHCF components. Tables containing the FTIR and Raman (λ = 532 nm) vibrational modes of the PLA. SEM images of the PLA/CB/rGO 3D-printed electrode. EDS mapping of PLA/CB/rGO/MnHCF electrode. TGA curves and XRD patterns of the MnHCF and PLA/CB/rGO/MnHCF materials. High resolution XPS of the MnHCF material: Mn 2p_3/2_ and Fe 2p_3/2_ regions. CV electrodes conditioning in 10 mol L^–1^ NaClO_4_ electrolyte solution. CV profile at 1 mV s^–1^ of the PLA/CB/rGO/MnHCF electrode in 10 mol L^–1^ NaClO_4_ before and after 3,500 GCD cycling. CV scan rate test of PLA/CB/rGO/MnHCF electrode and current contributions at all scan rates after long-term cycling in 10 mol L^–1^ NaClO_4_ electrolyte solution. SEM images of the PLA/CB/rGO/MnHCF electrode after 3,500 GCD cycles. EIS Nyquist diagram, XRD, FTIR, and Raman spectra of the PLA/CB/rGO/MnHCF electrode before and after 3,500 GCD cycles. High resolution XPS of the PLA/CB/rGO/MnHCF C 1s and O 1s regions before and after 3,500 GCD cycles (PDF)

## #

P.H.S.B.: Institute of Chemistry, University of Campinas, 13083–970, Campinas, SP, Brazil
