# Supplementary material for: Synergistic Charge Redistribution and Structural Evolution in Three-Dimensionally Printed Mn-Prussian Blue Analogue-Based Electrodes for Energy Storage
Source: ACS Nanosci Au. 2026 Mar 30;6(3):409–19. doi: 10.1021/acsnanoscienceau.5c00167 (PMC13281191; doi:10.1021/acsnanoscienceau.5c00167)

**Supporting information**

# Synergistic Charge Redistribution and Structural Evolution in 3D-Printed Mn-Prussian Blue Analogue-Based Electrodes for Energy Storage

*Pedro H. S. Borges,*[a,b,#] *Michele V. C. O. da Silva,*[a] *Jéssica S. Stefano,*[c] *Rafael R. Barreto,*[d] *Abner de Siervo,*[d] *Eduardo M. Richter,*[a] *Rodrigo A. A. Muñoz,*[a] *Juliano A. Bonacin,*[b] *Edson Nossol,*[a]*

[a] Institute of Chemistry, Federal University of Uberlândia, 38408-902, Uberlândia, MG, Brazil

[b] Institute of Chemistry, University of Campinas, 13083-970, Campinas, SP, Brazil

[c] Chemistry Technology Department, Federal University of Maranhão, 65080-805 São Luís, MA, Brazil

[d] Gleb Wataghin Institute of Physics, University of Campinas, 13083-970, Campinas, SP, Brazil

**Email:* enossol@ufu.br

**Keywords:** 3D printing, Fused deposition modeling, Energy storage, Prussian blue analogue, Electrochemical activation

**Figure S1.** SEM images of PLA **(a)**, CB **(b)**, rGO **(c)**, MnHCF **(d and e)**. MnHCF particles size histogram distribution **(f)**.

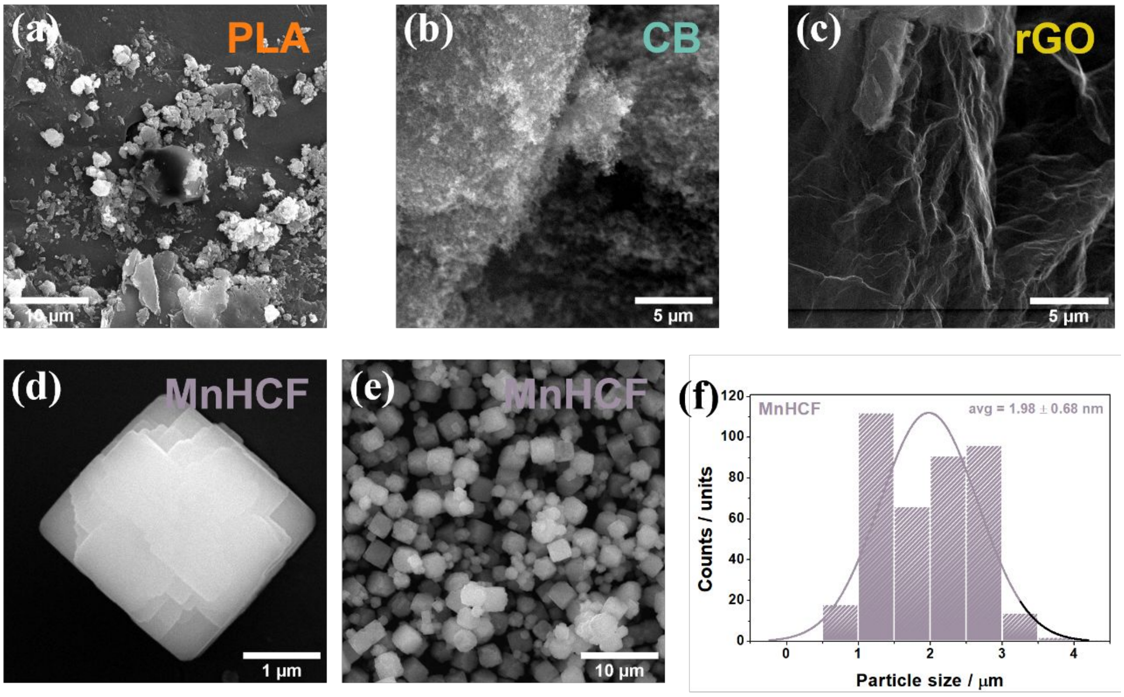


**Figure S2.** EDS spectrum of the MnHCF particles.

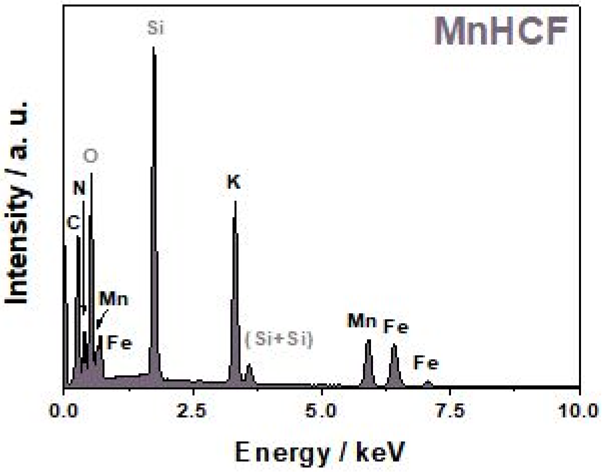

**Figure S3.** FTIR **(a)** and Raman **(b)** spectra of the individual components of the PLA/CB/rGO/MnHCF electrode.

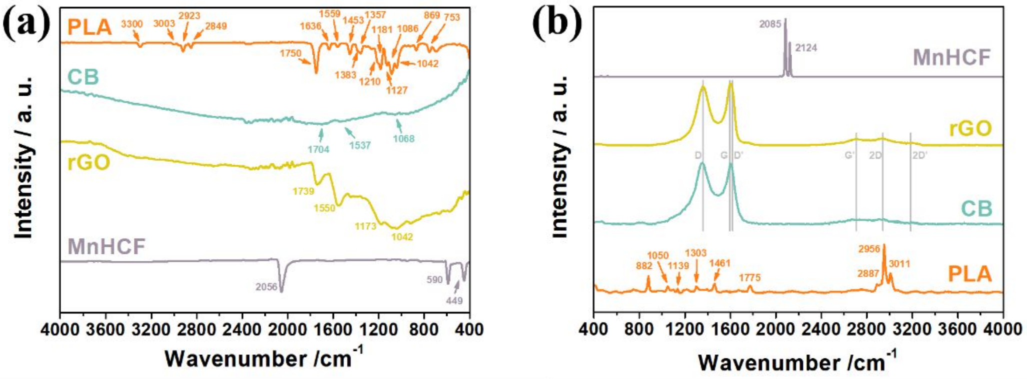


**Table S1.** Characteristic FTIR vibrational modes of PLA.

| Wavenumber / $cm^{-1}$ | Assignment |
|---|---|
| 753 | $\delta(C{=}O)$ |
| 869 | $\upsilon(C–COO)$ |
| 1042 | $\upsilon(C–O–C)$ |
| 1086 | $\upsilon(C–O–C)$ |
| 1127 | $\upsilon(C–CH_3)$ |
| 1181 | $\upsilon(C–O–C)$ |
| 1210 | $\upsilon(C{=}O)$ |
| 1357 | $\delta_s(CH_3)$ |
| 1383 | $\delta_s(CH)$ |
| 1453 | $\delta_{as}(CH_3)$ |
| 1559 | $\upsilon(C{=}CH_2)$ |
| 1636 | $\upsilon(C{=}C)$ |
| 1750 | $\upsilon(C{=}O)$ |
| 2849 | $\upsilon_s(C{=}CH_2)$ |

| | |
|---|---|
| 2923 | $\upsilon_s(C–CH_3)$ |
| 3003 | $\upsilon_{as}(C–CH_3)$ |
| 3300 | $\upsilon(O–H)$ |

**Table S2.** Raman vibrational modes of PLA (λ = 532 nm).

| **Wavenumber / cm$^{-1}$** | **Assignment** |
|---|---|
| 882 | $\upsilon(C–COO)$ |
| 1050 | $\upsilon(C–CH_3)$ |
| 1139 | $\rho_{as}(C–CH_3)$ |
| 1303 | $\delta_s(CH)$ |
| 1461 | $\delta_{as}(CH_3)$ |
| 1775 | $\upsilon(C=O)$ |
| 2887 | $\upsilon(CH_3)$ |
| 2956 | $\upsilon_s(CH_3)$ |
| 3011 | $\upsilon_{as}(CH_3)$ |

**Figure S4.** SEM images of the PLA/CB/rGO 3D-printed electrode.

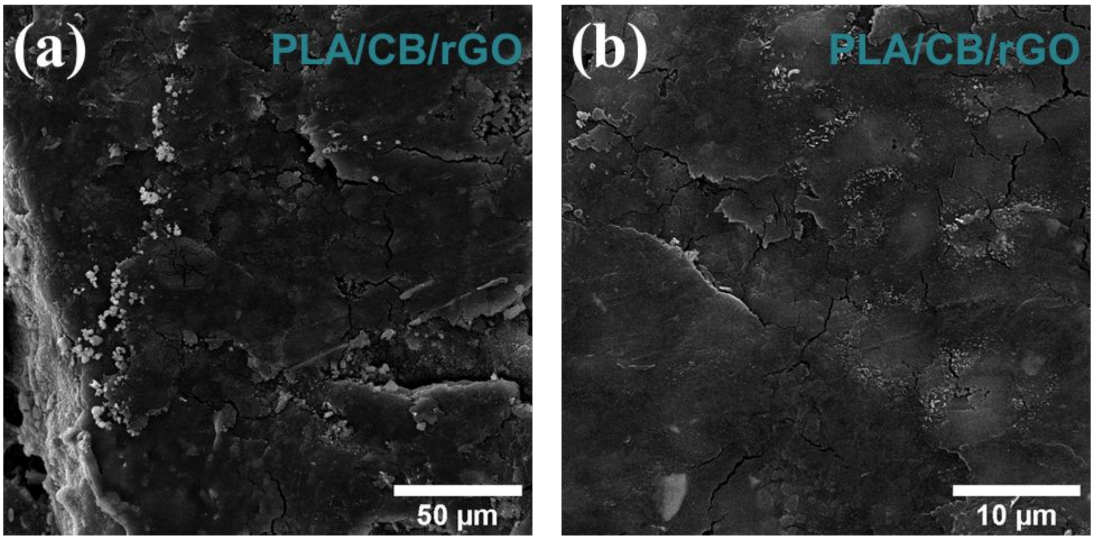

**Figure S5.** EDS mapping of the PLA/CB/rGO/MnHCF electrode.

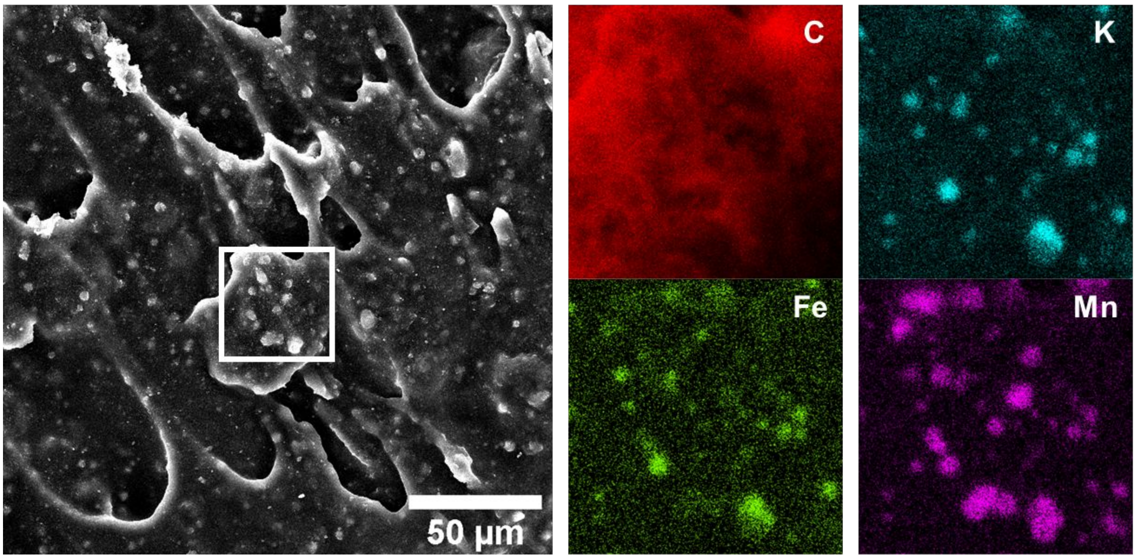


**Figure S6.** TGA curves **(a)** and XRD patterns **(b)** of the MnHCF and PLA/CB/rGO/MnHCF materials. High resolution XPS of the MnHCF material: Mn $2p_{3/2}$ **(c)** and Fe $2p_{3/2}$ **(d)** regions.

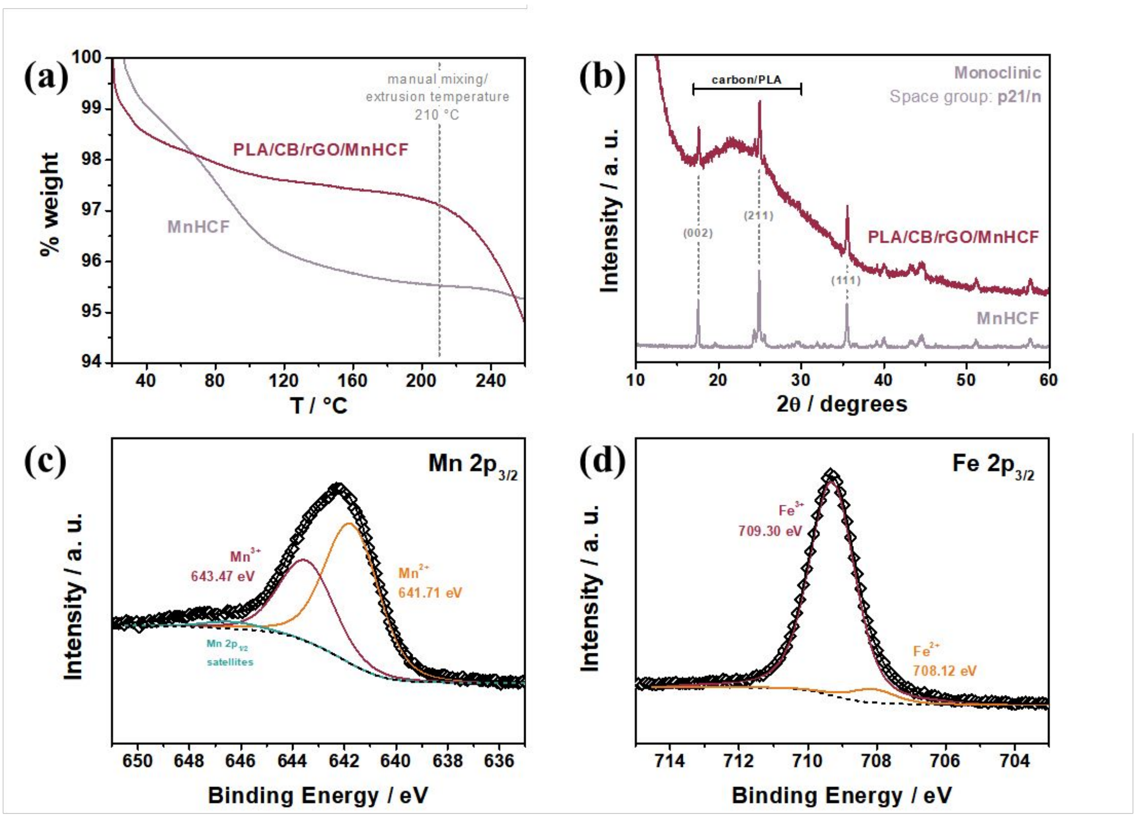

**Figure S7.** CV conditioning of the electrodes in 10.0 mol $L^{-1}$ $NaClO_4$ electrolyte solution.

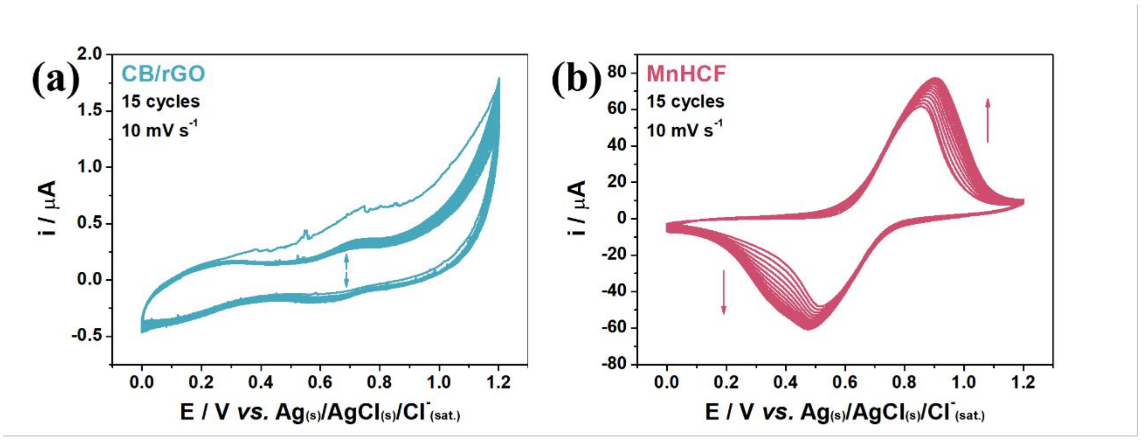


**Figure S8.** CV profile at 1 mV $s^{-1}$ of the PLA/CB/rGO/MnHCF electrode in 10.0 mol $L^{-1}$ $NaClO_4$ before and after 3,500 GCD cycles **(a)**. CV scan rate analysis of PLA/CB/rGO/MnHCF electrode **(b)** and corresponding current contributions across all scan rates **(c)** after long-term cycling in 10.0 mol $L^{-1}$ $NaClO_4$ electrolyte solution.

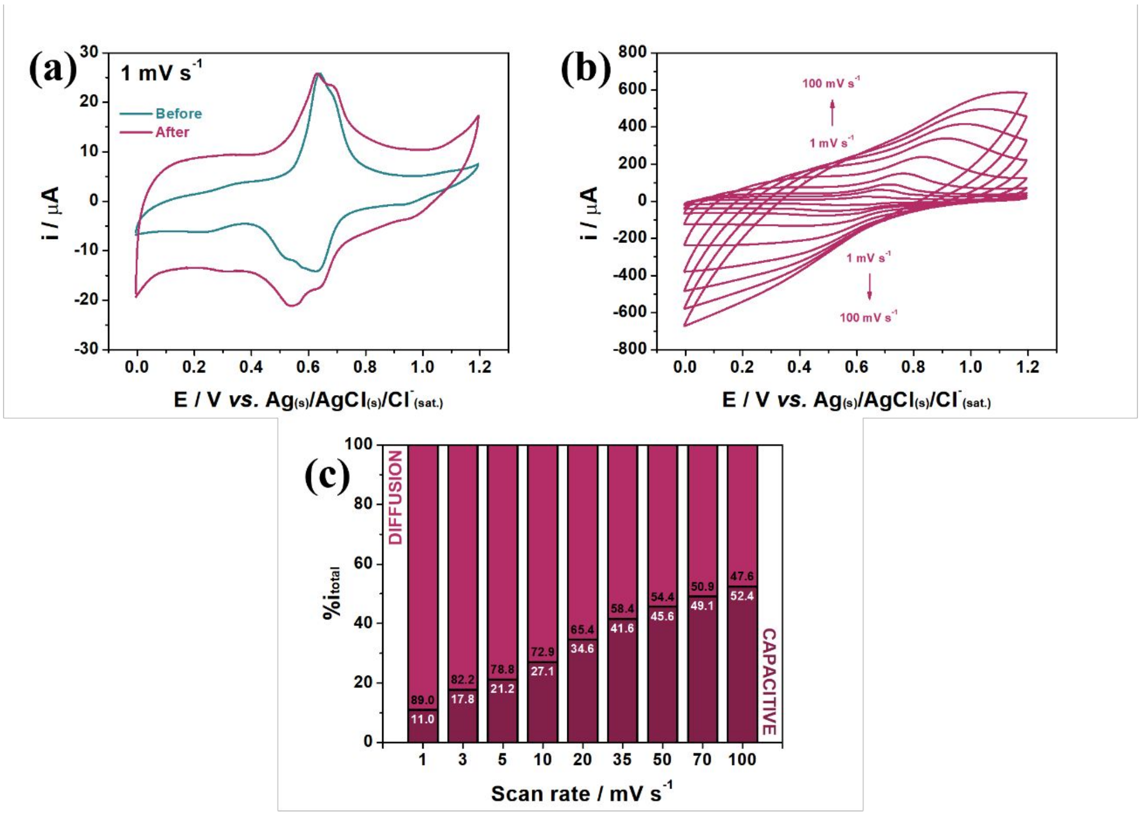

**Figure S9.** SEM images of the PLA/CB/rGO/MnHCF electrode after 3,500 GCD cycles.

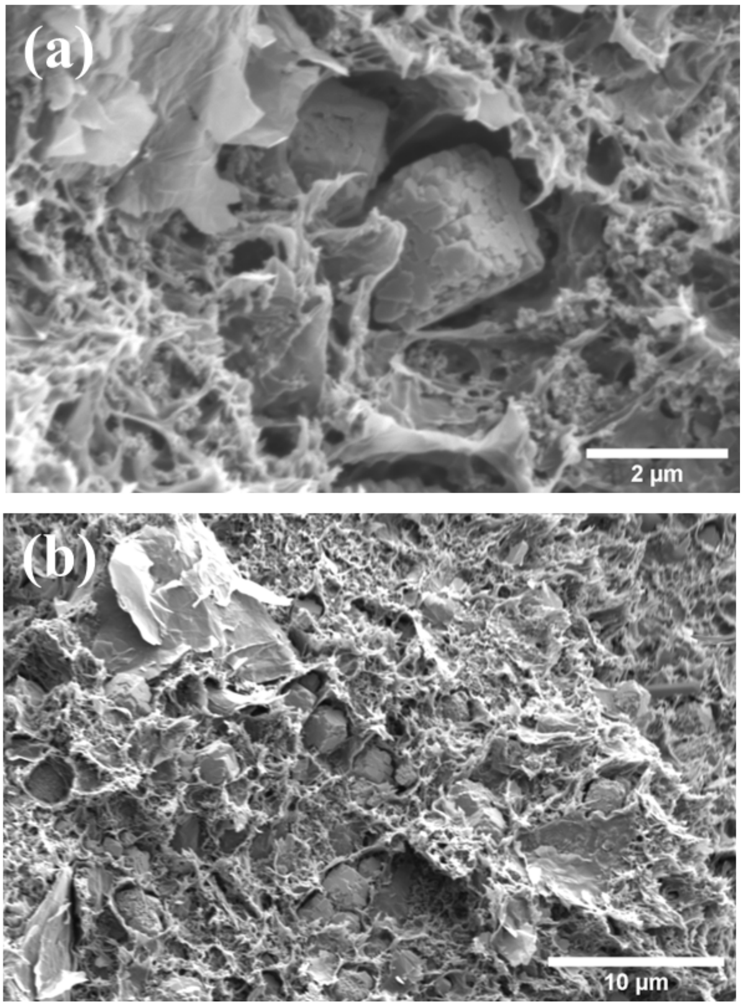

**Figure S10.** EIS Nyquist diagram **(a)**, XRD **(b)**, FTIR **(c)**, and Raman **(d)** spectra of the PLA/CB/rGO/MnHCF electrode before and after 3,500 GCD cycles.

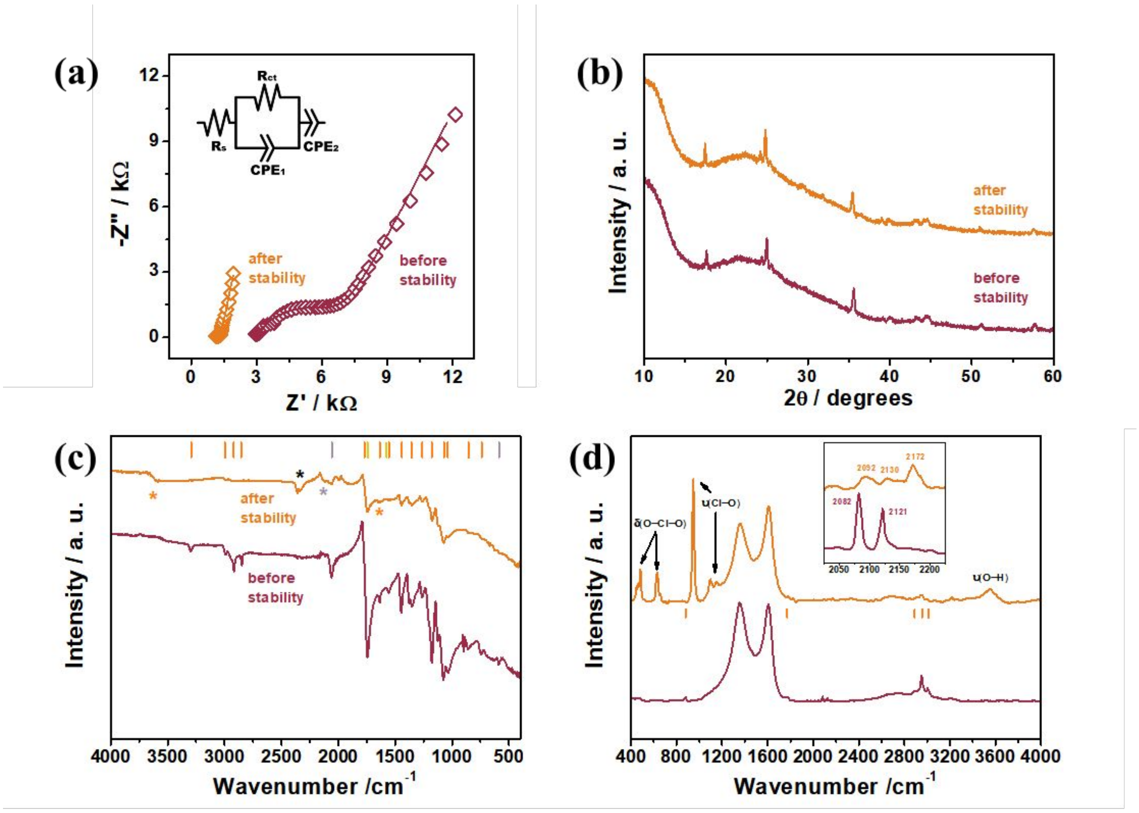

**Figure S11.** High resolution XPS of the PLA/CB/rGO/MnHCF C 1s **(a)** and O 1s **(b)** regions before and after 3,500 GCD cycles.

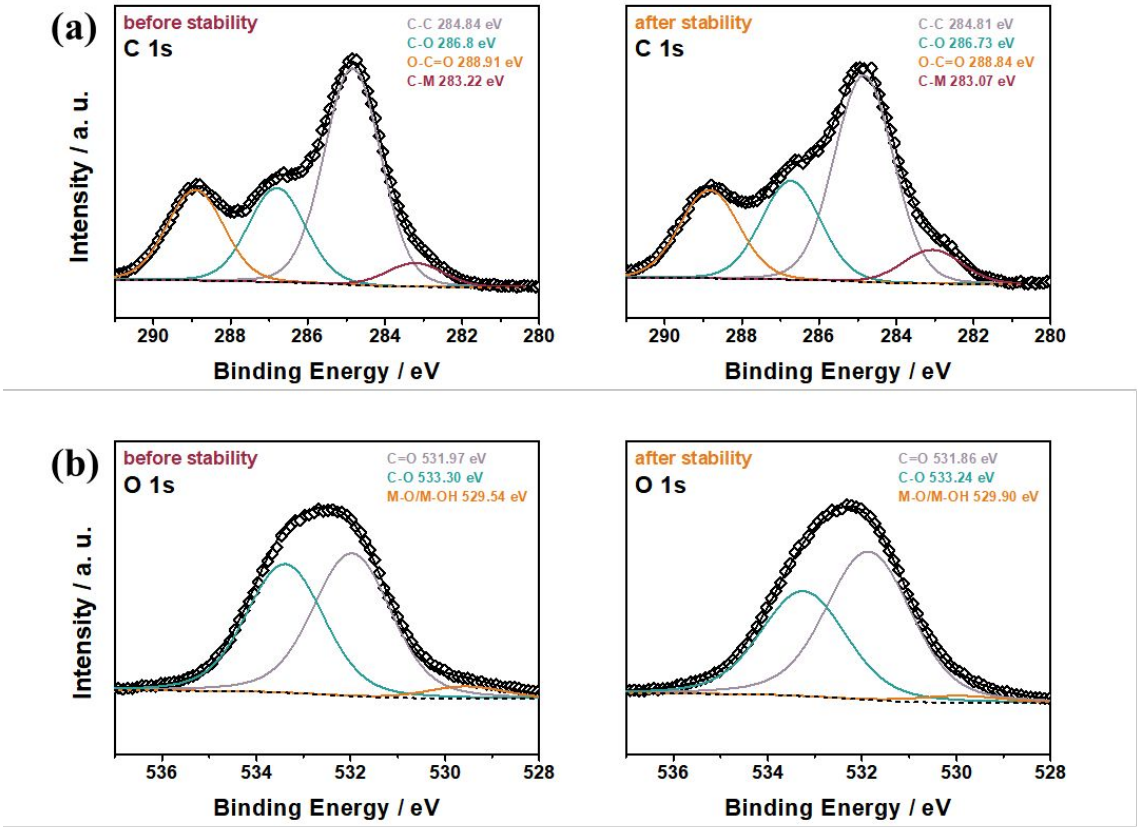

Supplement: Supplementary file 1 [file ng5c00167_si_001.pdf]
